# Interaction of the Transactivation Domain of B-Myb with the TAZ2 Domain of the Coactivator p300: Molecular Features and Properties of the Complex

**DOI:** 10.1371/journal.pone.0052906

**Published:** 2012-12-31

**Authors:** Ojore Oka, Lorna C. Waters, Sarah L. Strong, Nuvjeevan S. Dosanjh, Vaclav Veverka, Frederick W. Muskett, Philip S. Renshaw, Karl-Heinz Klempnauer, Mark D. Carr

**Affiliations:** 1 Department of Biochemistry, University of Leicester, Henry Wellcome Building, Leicester, United Kingdom; 2 Institut für Biochemie, Westfälische-Wilhelms-Universität, Münster, Germany; University of Crete, Greece

## Abstract

The transcription factor B-Myb is a key regulator of the cell cycle in vertebrates, with activation of transcription involving the recognition of specific DNA target sites and the recruitment of functional partner proteins, including the coactivators p300 and CBP. Here we report the results of detailed studies of the interaction between the transactivation domain of B-Myb (B-Myb TAD) and the TAZ2 domain of p300. The B-Myb TAD was characterized using circular dichroism, fluorescence and NMR spectroscopy, which revealed that the isolated domain exists as a random coil polypeptide. Pull-down and spectroscopic experiments clearly showed that the B-Myb TAD binds to p300 TAZ2 to form a moderately tight (K_d_ ∼1.0–10 µM) complex, which results in at least partial folding of the B-Myb TAD. Significant changes in NMR spectra of p300 TAZ2 suggest that the B-Myb TAD binds to a relatively large patch on the surface of the domain (∼1200 Å^2^). The apparent B-Myb TAD binding site on p300 TAZ2 shows striking similarity to the surface of CBP TAZ2 involved in binding to the transactivation domain of the transcription factor signal transducer and activator of transcription 1 (STAT1), which suggests that the structure of the B-Myb TAD-p300 TAZ2 complex may share many features with that reported for STAT1 TAD-p300 TAZ2.

## Introduction

**Figure 1:**
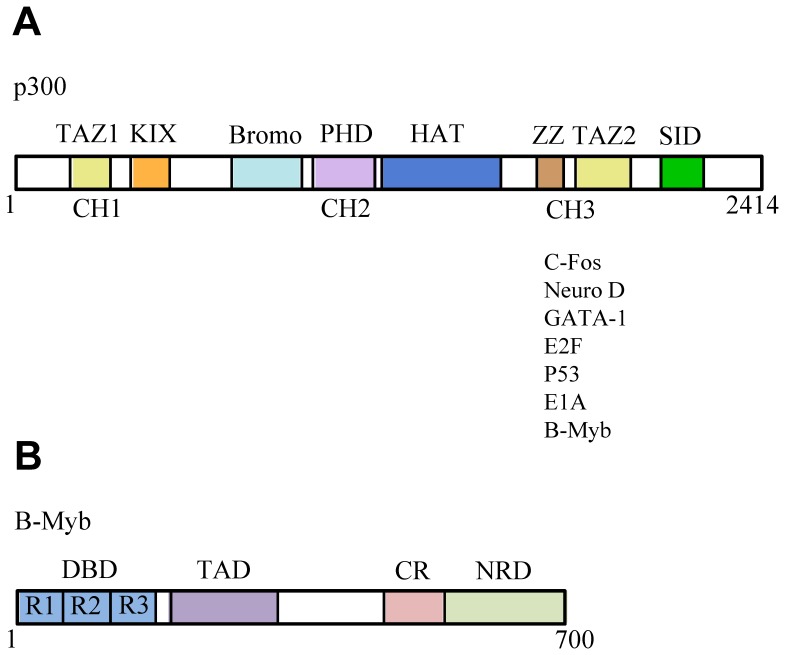
Schematic representations of the organisation of the functional regions and domains of human B-Myb and p300. Panel A shows the positions of functional domains in the transcriptional coactivator p300, as well as a partial list of proteins that bind to the CH3/E1A-binding region. Panel B illustrates the tripartite functional organisation of the B-Myb protein, which contains an N-terminal DNA binding region (DBD) formed by three highly homologous domains (R1, R2 and R3), a central transactivation domain (TAD), and towards the C-terminus a highly conserved region (CR) and negative regulatory domain (NRD).

In eukaryotes the regulation of transcription initiation involves coordinated interactions between a large number of proteins and complexes, including components of the basal transcription machinery, sequence-specific DNA-binding transcription factors such as B-Myb, coactivators and corepressors. Two key players in this process are the highly related proteins p300 and CBP (cAMP-response element binding (CREB)-binding protein), which are large transcriptional coactivators that contain a number of distinct structural and functional domains (figure 1A). p300 and CBP possess intrinsic histone acetyl transferase (HAT) and factor acetyl transferase (FAT) activities [1], [2], which indicate roles in the remodelling of chromatin and modification of transcription factors and coregulators. p300 and CBP also function as essential scaffold proteins, linking components of the basal transcription machinery to a multitude of transcription factors and coregulators [3], [4].

B-Myb is a member of the important Myb family of vertebrate transcription factors, which also includes A-Myb and c-Myb [5], and is a key regulator of the differentiation and proliferation of cells [6], [7], [8], [9]. In common with other members of the family B-Myb contains three functional regions (figure 1B), including a highly conserved, N-terminal DNA-binding domain (DBD) that recognises the consensus sequence PyAACG/TG (the Myb binding site (MBS), [10]). Adjacent to the DNA-binding region is a central transactivation domain (TAD), which shows no significant conservation across Myb proteins, whilst the regulatory C-terminus of the protein contains the highly conserved region (CR) and the negative regulatory domain (NRD, [11]).

The activity of B-Myb is tightly regulated by several types of post translational modification including acetylation and phosphorylation [12], [13], [14], [15], [16], [17]. For example, cyclin A/CDK2-mediated phosphorylation of B-Myb at the transition from G1 to S phase dramatically increases its transactivation potential, which is believed to involve the relief of repression by the C-terminal NRD [12], [13], [14], [16], [18]. A number of B-Myb regulated genes have now been identified in which activation of transcription involves the binding of B-Myb to consensus target sites (MBS) in their promoter or enhancer regions, leading to the recruitment of essential partner proteins such as the coactivators p300 and CBP [11], [17], [19], [20], [21], [22], [23], [24], [25], [26]. Previous studies have shown that c-Myb and A-Myb interact with the KIX domain of p300 and CBP via their central transactivation domain [27], [28], [29]. In contrast, the B-Myb transactivation region (residues 240–371) has been found to interact with the C-terminal E1A-binding region of p300, in particular, residues 1710–1891 [15], [17]. The precise molecular basis of the interaction and functional synergy between B-Myb and p300 remains to be determined and is the focus of the work reported here. In this communication we report detailed characterisation of the principal domains involved in B-Myb-p300 interactions and of the complex formed, including identification of the binding surface for the B-Myb TAD on the TAZ2 domain of p300.

## Materials and Methods

### Expression, Refolding and Purification of p300 TAZ2

The coding sequence of human p300 (1726–1812), corresponding to murine CBP TAZ2 [30], was obtained by PCR amplification and cloned into the *Nde*I and *Bam*HI sites of pET23a (Novagen). The recombinant plasmid was transformed into *Escherichia coli* (*E. coli)* BL21-CodonPlus® (DE3) RP cells (Stratagene) according to the manufacturer’s guidelines. Expression trials revealed that p300 TAZ2 was produced in *E. coli* as insoluble inclusion bodies. Unlabelled samples of p300 TAZ2 were produced from cells grown on LB medium. Uniformly ^15^N and ^15^N/^13^C-labelled samples were produced from cells grown on minimal medium, as described previously [31], [32]. After induction of p300 TAZ2 for 4 hours at 37°C, cells were harvested by centrifugation prior to lysis in buffer containing 50 mM Tris-HCl, 100 mM NaCl, 2 mM Dithiothreitol (DTT) and 0.5% (v/v) Triton X-100, pH 8.0, supplemented with 100 µg/ml Lysozyme (Sigma), 10 µg/ml DNase (Sigma), 5 mM MgCl_2_, an EDTA-free protease inhibitor tablet (Roche), and 100 µM phenylmethyl sulfonylfluoride (PMSF). Washed inclusion bodies were prepared from the cell lysate as described previously [31] and then solubilised in denaturing buffer (6 M-guanidine HCl, 20 mM Tris, 20 mM DTT, 100 mM NaCl buffer, pH 8.5) to give a final protein concentration of 0.4 mg/ml. Removal of the denaturant and refolding of the p300 TAZ2 was achieved by dialysis against a buffer containing 20 mM Tris, 100 mM NaCl, 200 µM ZnSO_4_ and 20 mM DTT, pH 8.5. The refolded TAZ2 then underwent a second dialysis against a buffer containing 20 mM Tris, 100 mM NaCl, 100 µM ZnSO_4_ and 2 mM DTT, pH 7.5 prior to being loaded onto a cation exchange column. The purified TAZ2 was eluted in 20 mM Tris, 1 M NaCl, 50 µM ZnSO_4_ and 2 mM DTT, pH 7.5 buffer and then purified to homogeneity by gel filtration chromatography on a Superdex 75 prep-grade column (Amersham Pharmacia) preequilibrated with buffer containing 20 mM Tris, 100 mM NaCl, 20 µM ZnSO_4_ and 5 mM DTT, pH 7.5. The purified TAZ2 was shown to be >95% pure by SDS-PAGE.

### Expression and Purification of the B-Myb TAD

GST-tagged mouse B-Myb TAD (residues 275–376) was expressed as a soluble fusion protein in *E. coli* and initially purified using glutathione agarose affinity chromatography [33]. B-Myb TAD was obtained after PreScission Protease (Amersham Pharmacia) cleavage of the GST-tag [34], [35]. Briefly, protein samples containing GST-tagged B-Myb TAD were dialysed against PreScission Protease cleavage buffer (50 mM Tris-HCl, 150 mM NaCl, 1 mM EDTA, 1 mM DTT, pH 7.0), prior to addition of PreScission Protease (10 U per mg of protein) and incubation for 16–20 hours at 4°C. The released GST and the GST-tagged PreScission protease were then removed by a second glutathione agarose affinity step, with the B-Myb TAD collected in the flow-through fractions. Homogenous B-Myb TAD was obtained after gel filtration chromatography on a Superdex 75 prep-grade column (Amersham Pharmacia), preequilibrated with buffer containing 20 mM Tris, 100 mM NaCl, 20 µM ZnSO_4_ and 5 mM DTT, pH 7.5. Purified B-Myb TAD was shown to be >95% pure by SDS-PAGE.

### Circular Dichroism Spectroscopy

CD data were acquired on a JASCO 715 spectropolarimeter at 25°C from protein samples of 8 to 20 µM in a 0.1 cm pathlength cell. Typically, spectra were recorded from 190 to 250 nm at a scan speed of 20 nm per minute, with each spectrum representing the average of 10 accumulations. Samples of p300 TAZ2 were prepared in a buffer containing 20 mM Tris, 100 mM NaCl, 2 mM DTT and 20 µM ZnSO_4_, pH 7.5, whilst samples of the B-Myb TAD were in a 25 mM sodium phosphate, 100 mM NaCl buffer at pH 7.0. Prior to secondary structure analysis, CD spectra were corrected for buffer absorbance and the raw data converted to molar CD per residue.

### Fluorescence Emission Spectroscopy

Intrinsic tryptophan fluorescence spectra were acquired on a Perkin Elmer LS50B luminescence spectrometer using a 1 cm path length cuvette, essentially as described previously [31]. For the B-Myb TAD, spectra were recorded from 3 µM samples in a 25 mM sodium phosphate, 100 mM NaCl buffer at pH 7.0. Samples of the B-Myb TAD (2.8 µM) in the presence of an approximate three-fold excess of p300 TAZ2 were prepared in a buffer containing 20 mM Bis-Tris, 100 mM NaCl, 2 mM DTT and 20 µM ZnSO_4_ buffer at pH 6.0. The B-Myb TAD-p300 TAZ2 samples were incubated for at least 1 hour at room temperature prior to recording spectra.

### GST Pull-down Assays

Pull-down assays between the GST-B-Myb TAD fusion protein and p300 TAZ2 were carried as follows. Initially, a 0.5 ml sample of 32.5 µM GST-B-Myb TAD was loaded onto a preequilibrated 0.5 ml glutathione agarose column and washed with 5 column volumes of binding buffer (20 mM Bis-Tris, 100 mM NaCl, 2 mM DTT, 20 µM ZnSO_4_, pH 7.2). An equivalent 0.5 ml sample containing a slight molar excess of p300 TAZ2 was then loaded onto the column and washed with 8 column volumes of binding buffer to remove unbound proteins. Bound proteins were eluted by the addition of binding buffer containing 10 mM reduced glutathione and the elution fractions were analysed by SDS-PAGE, with the relative staining intensity of the coomassie stained bands determined using the program TINA (Isotopenmessgerate GmbH). Identical control pull-down assays were performed between GST and p300 TAZ2.

### NMR Spectroscopy

NMR spectra were acquired from 0.35 ml samples of 0.3 mM p300 TAZ2 or 0.15 mM B-Myb TAD, in a 20 mM Bis-Tris, 100 mM NaCl, 5 mM DTT, 20 µM ZnSO_4_ and 0.02% (w/v) NaN_3_ buffer (pH 5.8), containing 10% D_2_O. All NMR data were acquired at 25°C on 600 MHz Bruker Avance or DRX systems. The two-dimensional (2D) and three-dimensional (3D) spectra recorded to obtain sequence-specific assignments for p300 TAZ2 were as follows: ^15^N/^1^H HSQC [36] ^15^N/^13^C/^1^H HNCACB [37], CBCA(CO)NH [38] and HNCO [39]. Typical acquisition times in F_1_ and F_2_ for the 3D experiments were 20 ms for ^15^N, 9 ms for ^13^C (25 ms for HNCO), and an acquisition time of 60 ms in F3 (^1^H). The majority of the 3D spectra were collected over ∼90 hours and the ^15^N/^1^H HSQC spectra over about 45–60 minutes. The WATERGATE method [40] was used to suppress the water signal when required. The NMR data were processed using Topspin (Bruker Biospin Ltd) with linear prediction used to extend the effective acquisition times by up to 1.5 times in ^15^N. Spectra were analysed using the Sparky package (T. D. Goddard and D. G. Kneller, SPARKY 3, University of California, San Francisco).

NMR binding experiments were carried out essentially as reported previously [41]. Briefly, ^15^N/^1^H HSQC spectra of ^15^N-labelled p300 TAZ2 (100 µM) were acquired in the presence and absence of either 100 or 200 µM unlabelled B-Myb TAD, to identify the changes in the positions of backbone amide signals induced by B-Myb TAD binding. The minimal shift approach [42], [43], [44] was used to determine the changes in the positions of p300 TAZ2 signals resulting from the interaction with the B-Myb TAD.

### Sequence-specific NMR Assignments and Secondary Structure Determination

Sequence-specific backbone resonance assignments were obtained for p300 TAZ2 from the identification of intra- and inter-residue connectivities in ^15^N/^13^C/^1^H HNCACB, CBCA(CO)NH and HNCO spectra. The chemical shift index [45] and TALOS [46] programs were used to determine the positions of elements of secondary structure from the chemical shift data.

## Results

### Spectroscopic Characterisation of the B-Myb TAD

**Figure 2:**
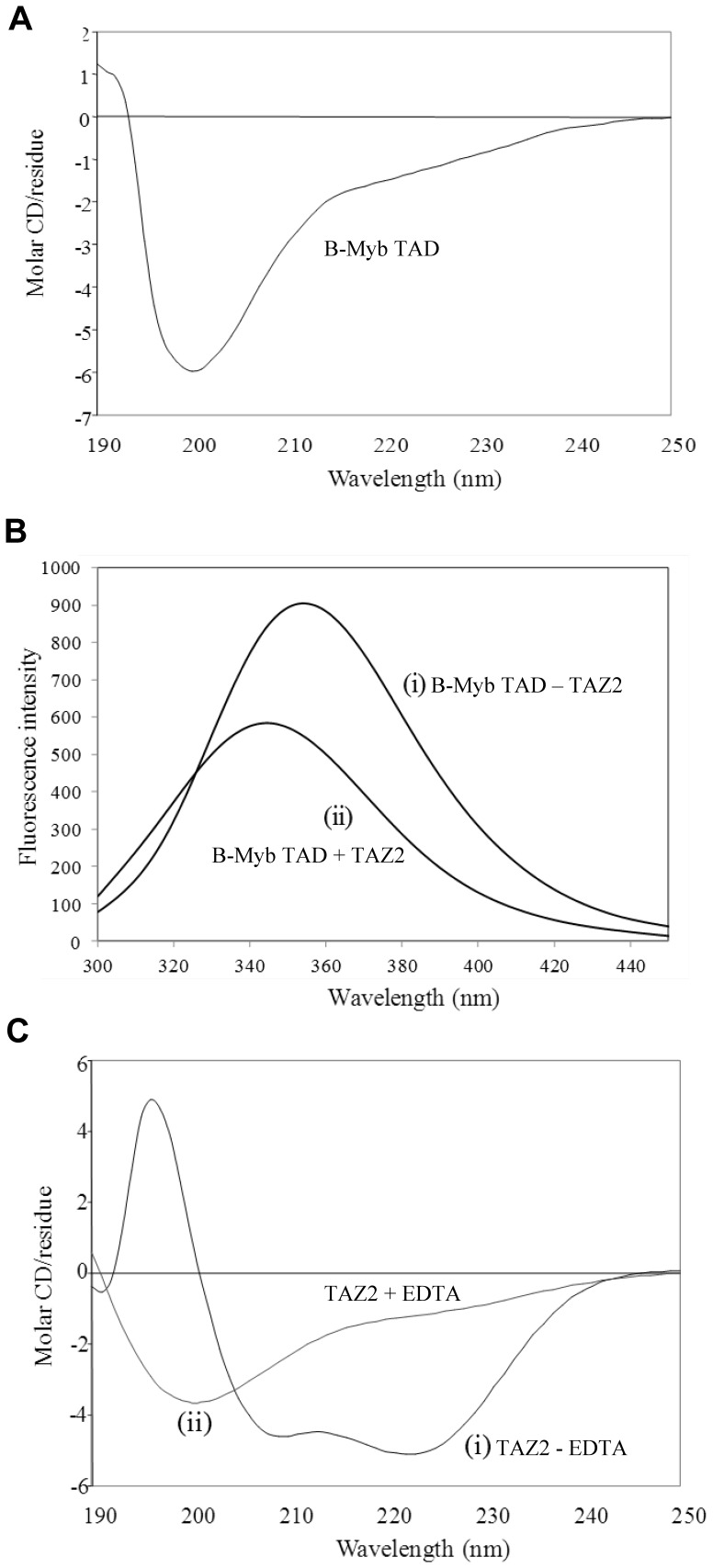
Far UV circular dichroism analysis of the B-Myb TAD and p300 TAZ2 domain. Panels A illustrates a typical far UV circular dichroism (CD) spectra obtained for the B-Myb TAD. Panel B shows representative intrinsic tryptophan fluorescence emission spectra obtained for the B-Myb TAD in the absence (i) and presence (ii) of an approximately three-fold molar excess of p300 TAZ2. In panel C far UV CD spectra of TAZ2 are shown in the absence (i) and presence (ii) of a molar excess of EDTA over Zn^2+^ ions.

A typical far-UV CD spectrum obtained for the B-Myb TAD is shown in figure 2A and is characterised by a large negative peak at approximately 200 nm, which together with the overall profile of the spectrum strongly suggests that the isolated B-Myb TAD forms a random coil polypeptide, with very little if any regular secondary structure. The B-Myb TAD contains two tryptophan residues (W293 and W323). The intrinsic fluorescence spectrum of B-Myb TAD (figure 2B (i)) is also typical of that expected for an unstructured polypeptide, with an emission maximum at 354 nm corresponding to tryptophan side chains that are fully exposed to the aqueous solvent. The unstructured nature of the isolated B-Myb TAD was confirmed by 1D ^1^H NMR spectra of the protein, which showed no signals shifted from the random coil positions (data not shown).

### Spectroscopic Characterisation of p300 TAZ2

Figure 2C (i) shows a typical far UV CD spectrum obtained for the p300 TAZ2 domain. This is characterised by two large negative peaks at approximately 208 and 222 nm, which indicate a predominantly helical structure. Previous work has shown that the equivalent region of the very closely related TAZ2 domain of CBP contains five helices, with the tertiary structure of the domain stabilised by the coordination of three zinc ions [30]. To assess the importance of Zn^2+^ binding for p300 TAZ2, samples were incubated with EDTA and analysed by CD (figure 2C (ii)), which resulted in a far-UV spectrum typical of a random coil polypeptide. This clearly indicates that the p300 TAZ2 domain also requires Zn^2+^ ions to adopt a stable folded structure. Similar results were recently published for a non-native construct of human p300 TAZ2 (residues 1723–1812) in which the non-zinc coordinating cysteines had been mutated to alanine residues [47].

**Figure 3:**
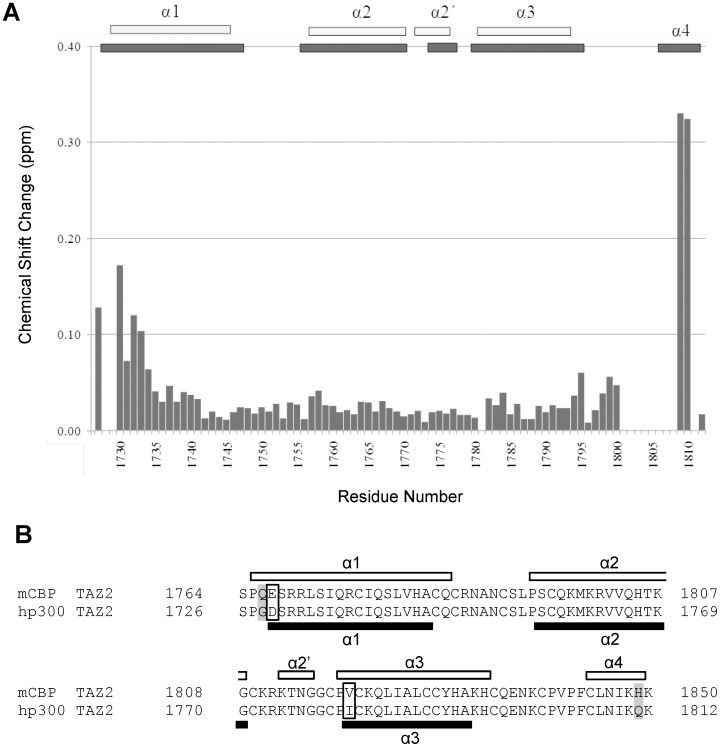
Comparison of NMR assignments and secondary structures for the TAZ2 domains of CBP and p300. Panel A summarises the combined differences in backbone amide (^15^N and ^1^H), CO and Cα chemical shifts for equivalent residues in the TAZ2 domains of CBP and p300. To compensate for the increased chemical shift range of ^15^N and ^13^C compared to ^1^H, the combined change was calculated as (Δ^1^HN+(Δ^15^N × 0.2)+(Δ^13^Cα × 0.1)+(Δ^13^CO × 0.35))/4. In a very few cases where some of the chemical shifts were not available, the sum of the chemical shift changes was divided by the number of available shift differences. Panel B shows an alignment of the very closely related TAZ2 sequences from CBP and p300. Conservative substitutions are highlighted in an open box and non-conservative highlighted in grey. The black bars shown indicate the positions of the helices in CBP TAZ2 [30], whilst the white bars represent the positions of the helices in p300 TAZ2, which were identified by analysis of the backbone resonance assignments using the chemical shift index method [45].

^15^N/^1^H HSQC spectra obtained from uniformly ^15^N labelled samples of p300 TAZ2 show many well dispersed peaks, indicative of the majority of residues forming a folded globular domain. Analysis of a series of triple-resonance NMR spectra acquired from samples of p300 TAZ2 allowed essentially complete backbone resonance assignments (N, NH, Cα, Cβ and CO) to be made for p300 TAZ2. The structural implications of this information were assessed using the programs CSI and TALOS, which resulted in the identification of four helical regions in p300 TAZ2 comprising residues Asp^1729^-Ala^1745^ (α1), Ser^1757^-Gly^1770^ (α2), Lys^1772^-Asn^1776^ (α2′) and Ile^1781^-Ala^1793^ (α3). TALOS identified the helical regions to comprise residues Gly^1728^-Gln^1747^ (α1), Pro^1756^-Gly^1770^ (α2), Arg^1773^-Asn^1776^ (α2′) and Ile^1781^-Lys^1794^ (α3). To date no chemical shift assignments have been reported for the isolated p300 TAZ2 domain, however, with the exception of the regions located near the N- and C-termini, the chemical shifts of p300 TAZ2 are very similar to those previously determined for CBP TAZ2 (figure 3A, [30]), suggesting that the domains will adopt very similar secondary and tertiary structures. Importantly, virtually identical Cβ chemical shifts were observed for the eleven of thirteen cysteine residues that we were able to obtain assignments for (average difference 0.09±0.08 ppm). The Cβ chemical shift can be used to confirm whether cysteine residues are coordinating a zinc ion, as this results in a significant downfield shift [30], [48]. Unfortunately, due to the absence of histidine ring assignments we were unable to confirm the identity of the three zinc-coordinating histidine residues, however, given the close similarity of the cysteine Cβ chemical shifts it is very likely that our construct contains three correctly coordinated zinc ions.

### B-Myb TAD-p300 TAZ2 Complex Formation

**Figure 4:**
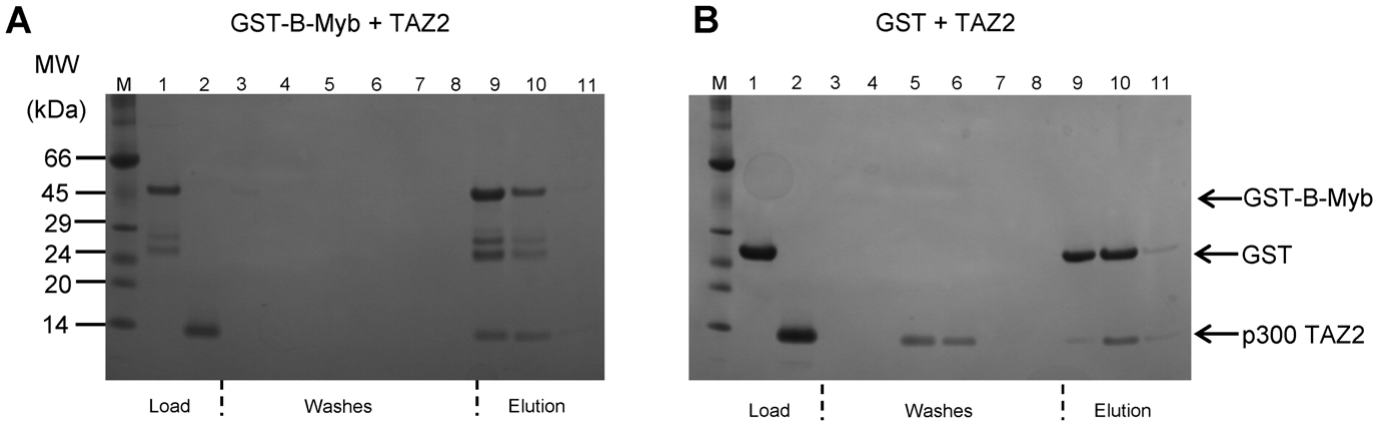
Binding of the B-Myb TAD to the TAZ2 domain of p300. The SDS-PAGE gel shown in panel A illustrates the analysis of a typical pull down experiment using immobilised GST-B-Myb TAD as bait and a slight excess of p300 TAZ2 as the potential interaction partner. p300 TAZ2 was loaded onto a glutathione agarose that had been preloaded with a 0.5 ml sample of GST-B-Myb TAD (32.5 µM), the column was washed with 8 column volumes of binding buffer prior to elution. Lane M contains molecular weight markers, lane 1 contains the GST-B-Myb load, lane 2 the p300 TAZ2 load, lanes 3–8 are consecutive washes, and lanes 9 to 11 are consecutive elution fractions. The SDS-PAGE gel shown in panel B shows the results of a control pull-down assay in which p300 TAZ2 was loaded onto the column in the presence of GST alone. The samples loaded on the gel are identical to those described in panel A, except lane 1 contains the GST load.

Pull-down assays using GST-tagged B-Myb TAD as bait were used to probe potential interactions between the B-Myb TAD and p300 TAZ2. The SDS-PAGE gel shown in figure 4A illustrates the results of a typical pull-down assay conducted at a 1∶1 molar ratio of GST-B-Myb TAD:p300 TAZ2, which demonstrates that the p300 TAZ2 domain binds to the immobilized GST-B-Myb fusion protein. In control experiments where an equivalent amount of p300 TAZ2 was loaded in the presence of GST alone the majority of the protein came through the column in wash fractions, however some p300 TAZ2 protein did bind to the column, as shown in figure 4B. Semi-quantitative analysis of the staining intensities observed for the p300 TAZ2 loaded compared to the protein retained by columns containing either immobilized GST-tagged B-Myb or GST alone, clearly indicates that essentially all the loaded TAZ2 binds tightly to an equimolar amount of GST-B-Myb whereas only ∼45% is bound by the column containing GST. Further comparison of the representative SDS-PAGE gels shown suggests that the p300 TAZ2 does not co-elute with GST, but rather elutes slightly later, perhaps indicative of an interaction between the column matrix and the TAZ2 domain. In agreement with this finding similar results were obtained when the p300 TAZ2 was loaded onto the column in the absence of GST (data not shown). Despite the presence of this interaction between the matrix and p300 TAZ2 it is clearly evident that in the presence of GST-B-Myb TAD substantially more TAZ2 binds to the column. In addition, the elution profile of p300 TAZ2 changes in the presence of GST-B-Myb TAD, such that the two domains co-elute, providing clear evidence of an interaction between B-Myb TAD and p300 TAZ2.

In order to confirm the pull-down assay results we recorded intrinsic tryptophan fluorescence spectra of B-Myb TAD in the presence and absence of an approximate three-fold excess of p300 TAZ2. The TAZ2 domain of p300 contains no tryptophan residues and exhibits no significant intrinsic fluorescence. In contrast, the B-Myb TAD contains two central tryptophan residues (Trp^293^ and Trp^323^), with the potential to show significant changes in fluorescence on TAZ2 binding. The addition of an approximate three-fold excess of p300 TAZ2 to samples of the B-Myb TAD resulted in a shift in the tryptophan fluorescence maximum from 354 to 344 nm, as shown in figure 2B, which clearly reflects a change in the tryptophan environment on formation of the B-Myb TAD-TAZ2 complex. This also suggests that the region encompassing one or both tryptophan residues in the B-Myb TAD adopts a folded conformation on binding to the TAZ2 domain. Unfortunately, given the low extinction coefficient of p300 TAZ2 (∼1490 M^−1^ cm^−1^) and the required presence of DTT in the buffers it was not possible to accurately determine the protein concentration of TAZ2 [49]. This precludes the possibility of using fluorescence titration data to reliably determine the affinity or stoichiometry of the complex.

**Figure 5:**
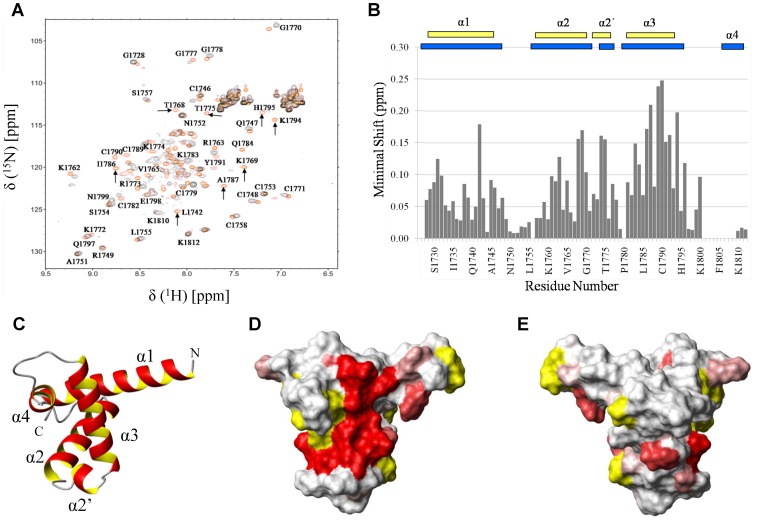
Identification of the B-Myb TAD binding site on p300 TAZ2. Panel A shows an overlay of two ^15^N/^1^H HSQC spectra of ^15^N labeled p300 TAZ2 (100 µM) acquired in the absence (red) or presence of equimolar unlabelled B-Myb TAD (black). The arrows highlight a number of TAZ2 signals which show significant shifts on interaction with the B-Myb TAD. Panel B contains a histogram summarizing the minimal chemical shift changes observed for backbone amide signals of p300 TAZ2 on binding to B-Myb TAD, with gaps corresponding to proline residues (1727, 1756, 1780, 1802 and 1804) or non-observable backbone amides. The combined amide proton and nitrogen chemical shift difference (Δδ) was defined according to the calculation 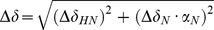 where α_N_ is a scaling factor of 0.2 required to account for differences in the range of amide proton and nitrogen chemical shifts. The reported positions of the helices in CBP TAZ2 (blue bars, [30]), together with those determined for p300 TAZ2 (yellow bars), are shown above the histogram. Panel C shows a ribbon representation of the backbone structure of the TAZ2 domain of CBP [30] and panel D a contact surface view in the same orientation. In panel E the surface view of CBP TAZ2 has been rotated by 180° about the y axis. The contact surfaces have been coloured according to the magnitude of the minimal shifts induced in backbone amide resonances of equivalent residues in p300 TAZ2 by binding of the B-Myb TAD. Residues that showed a minimal shift change of less than 0.075 ppm are shown in white, over 0.15 ppm in red, and between 0.075 and 0.15 ppm are coloured according to the level of the shift on a linear gradient between white and red. No chemical shift perturbation data could be obtained for the residues shown in yellow.

To confirm the specificity of the B-Myb TAD-p300 TAZ2 interaction, and to identify the residues of TAZ2 involved in interactions with the B-Myb TAD, NMR spectroscopy was used to monitor changes in the backbone amide signals of p300 TAZ2 induced by complex formation. Figure 5A shows typical ^15^N/^1^H HSQC spectra obtained from samples of ^15^N-labelled p300 TAZ2 (100 µM) in the absence (red) and presence (black) of an equivalent amount of unlabelled B-Myb TAD. The addition of the B-Myb TAD results in significant shifts in the positions of a subset of signals, as well as substantial line broadening leading to a loss of a few peaks. Addition of a second molar equivalent of B-Myb TAD resulted in further line broadening and loss of the majority of the peaks (data not shown). The extent of the line broadening observed required acquisition times of about 12 hours to obtain acceptable ^15^N/^1^H HSQC spectra for samples containing equivalent molar amounts of ^15^N-labelled p300 TAZ2 and unlabelled B-Myb TAD and precludes any possibility of obtaining assignments for either protein in the B-Myb TAD-p300 TAZ2 complex. The effects seen are characteristic of the formation of a protein complex in intermediate exchange on the NMR time scale, which is reflected in the fact that HSQC spectra of the complex obtained at 600 MHz were significantly better than spectra acquired at 800 MHz. The minimal chemical shift changes observed for the backbone amide signals of the TAZ2 domain induced by the binding of B-Myb TAD are summarised in the histogram shown in figure 5B, The minimal shifts of signals from a small number of non-proline residues (Ser^1726^, Cys^1801^, Val^1803^, Phe^1805^, Cys^1806^, Leu^1807^, Asn^1808^ and Ile^1809^) in TAZ2 could not be determined due to missing backbone amide resonances in ^15^N/^1^H HSQC spectrum of the complex.

## Discussion

### B-Myb TAD

Previous reports have identified the poorly characterised, central transactivation region of B-Myb as the binding site for several functional partner proteins [15], [50]. We have expressed the region corresponding to the B-Myb transactivation domain (residues 275–376) in *E. coli* as a GST fusion protein and characterised the properties of the purified B-Myb TAD using a range of spectroscopic techniques. CD and NMR spectra of the B-Myb TAD clearly show that it forms a random coil polypeptide, with no regular secondary or tertiary structure. This is consistent with the observed tryptophan fluorescence emission maximum of 354 nm, which indicates that the two tryptophan side chains are fully exposed to the aqueous environment.

**Figure 6:**
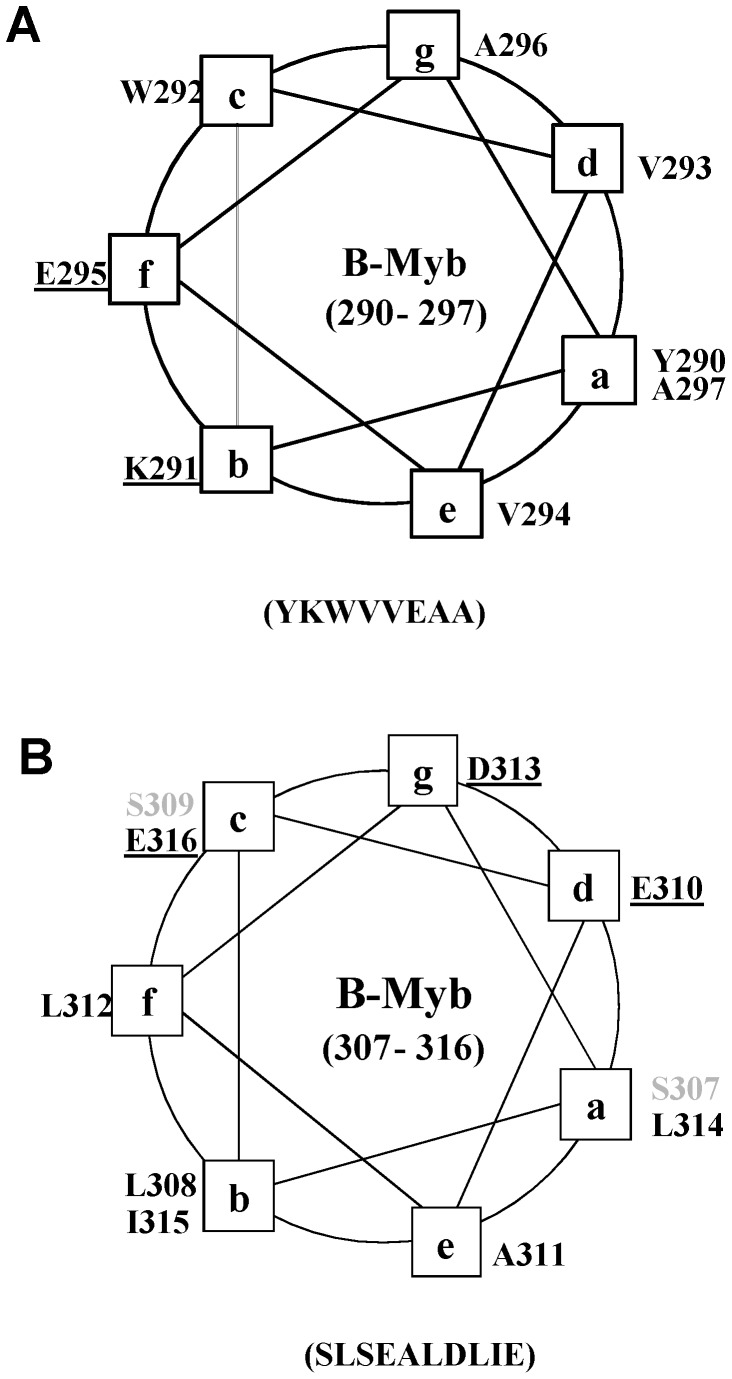
Potential amphipathic helices in the B-Myb TAD. Panels A and B show helical wheel representations of the regions of the B-Myb TAD predicted to form amphipathic helices, charged residues are underlined and polar residues shown in italics. The positions of the helical regions were predicted using the programme PSIPRED [71], [72].

The random coil nature of the B-Myb TAD is not entirely unexpected, as this region contains a fairly high proportion of polar and charged amino acid residues (Gln/Asn 10%, Ser/Thr 15%, Asp/Glu 18%, Lys/Arg 6%), as well as many proline residues (11%), which are features associated with intrinsically disordered regions and are characteristics of many transcriptional activation domains [51], [52]. Unstructured TADs have been reported for a number of transcription factors, including the kinase-inducible activation domain (KID) of CREB [53], the C-terminal activation domain of Hif-1α [54], [55], the activation domains of STAT-1 and 2 [56] and the activation domain of the glucocorticord receptor [57]. Many transcriptional regulators are known to contain similar unstructured regions that adopt well defined conformations on binding to functional partner proteins [32], [54], [56], [58], [59], [60], [61]. The intrinsically disordered nature of the B-Myb TAD may confer several thermodynamic and functional advantages, including the ability to bind to a diverse range of partner proteins with high specificity but moderate affinities, consistent with the formation of transient regulatory complexes [50], [62]. Previous studies with intrinsically disordered TADs have identified regions with the tendency to form amphipathic helices as important interaction sites [58], [63]. Secondary structure predictions for the B-Myb TAD suggest the potential to form two short helices between residues Tyr^290^ and Ala^297^ (YKWVVEAA) and residues Ser^307^ and Glu^316^ (SLSEALDLIE). Helical wheel analysis of these regions reveals that the helices formed would be amphipathic (figure 6) and contain extensive hydrophobic faces for interaction with functional partner proteins such as p300. Interestingly, the two potential helices are contained within a 47 residue region of B-Myb (Pro^289^-Ser^335^ in mouse) that is highly conserved between human, mouse, chicken and zebrafish (figure S1, 32% sequence identity and 26% sequence similarity).

### p300 TAZ2

The TAZ2 domain of p300/CBP is an important protein-protein interaction site and has been reported to bind a multitude of functional partners involved in the regulation of transcription, including the adenovirus E1A oncoprotein and p53 [61], [64], [65], [66]. The p300 TAZ2 domain was produced as inclusion bodies in *E. coli* and refolded by dialysis in the presence of an ∼5 fold excess of zinc ions. CD and NMR spectra of the isolated p300 TAZ2 domain clearly show that it forms a folded globular domain, which is stabilised by the binding of zinc ions. The far UV CD spectra also indicate that the domain contains a large proportion of regular helical structure.

Comparison of the backbone resonance assignments obtained for the p300 TAZ2 domain with those reported for the highly homologous domain in CBP (figure 3A) strongly suggest that p300 TAZ2 adopts a very similar structure to that reported for CBP TAZ2. This is further supported by comparison of the position of the helical regions in p300 TAZ2 with those described for CBP TAZ2, which reveals very close similarities; except for the last short helix in CBP TAZ2 (Cys^1844^-His^1849^, figure 3B), which appears to be absent in p300 TAZ2. The absence of this final C-terminal helix in p300 TAZ2 is reflected by the significant chemical shift differences in this region (figure 3A). Interestingly, NMR backbone amide signals are missing for residues Val^1803^, Phe^1805^, Cys^1806^, Leu^1807^, Asn^1808^ and Ile^1809^ in p300 TAZ2, which suggests conformational heterogeneity in this C-terminal region. During the preparation of this paper the isolated structure of an extended human p300 TAZ2 (residues 1723–1836) construct was published (PDB code 3IO2) [67]. This construct contains an extended C-terminal helix composed of residues 1806–1832. It seems likely that the additional residues are required to stabilize the final C-terminal helix of the isolated p300 TAZ2 domain, which explains the conformational heterogeneity we observed in this region of our shorter construct. A similar longer helix is also observed in the structure of the p300 TAZ2- myocyte enhancer factor 2 (MEF2) complex [68].

### B-Myb TAD-p300 TAZ2 Interaction

A number of reports have highlighted the importance of the B-Myb transactivation domain (TAD) in functional interactions with partner proteins, including the interaction with p300/CBP [15], [17], which results in the synergistic activation of transcription. Previous attempts to identify the B-Myb-binding site on p300 have localised the site of interaction to the E1A-binding region, which encompasses the ZZ and TAZ2 domains [15], [17]. The GST pull-down and fluorescence results reported here clearly demonstrate an interaction between the isolated B-Myb TAD and TAZ2 domain, which strongly suggests that the TAZ2 domain contains the principal site of B-Myb binding. The shift in the tryptophan fluorescence maximum of the B-Myb TAD from 354 to 344 nm, induced by TAZ2 binding, clearly suggests coupled folding and binding of the B-Myb TAD, as observed for other transcriptional regulators [32], [54], [56], [58], [60], [61].

Changes in ^15^N/^1^H HSQC spectra of p300 TAZ2 induced by B-Myb binding provide clear evidence that B-Myb TAD binds to a specific region on the surface of TAZ2. The significant broadening of selected p300 TAZ2 resonances on complex formation implies intermediate exchange on the NMR time scale between the free and bound species, which is consistent with a K_d_ in the low micromolar range (1–10 µM). Similar relatively low affinity interactions have recently been reported for a number of protein complexes involved in transcriptional and translational regulation [65], [69], [70], which is consistent with the requirement to form transient or dynamic complexes for effective regulation of these processes. The TAZ2 signals most perturbed by B-Myb TAD binding correspond to residues Arg^1731^, Leu^1742^, Arg^1763^, Thr^1768^, Lys^1769^, Gly^1770^, Lys^1774^, Thr^1775^, Gln^1784^, Ile^1786^, Ala^1787^, Cys^1789^, Cys^1790^, Tyr^1791^, His^1792^, Lys^1794^ and Cys^1796^ (figure 5B). The locations of the perturbed residues were mapped onto the surface of both the isolated CBP TAZ2 domain ([30]: PDB code 1F81: figure 5 panels D & E) and the isolated p300 TAZ2 domain ([67]: PDB code 3IO2: figure S2). The majority of the shifted residues are located at the C- terminus of α2, in α2′, and on the exposed face of α3. These residues form a large patch on the surface of TAZ2 (∼1200 Å^2^, figure 5), which is consistent with forming a contiguous binding surface for the B-Myb TAD, rather than reflecting a conformational change induced by B-Myb TAD binding. Interestingly, a number of residues for which we were unable to obtain chemical shift mapping data (Pro^1780^ and Cys^1801^-Ile^1809^), including several that appear to be in conformational exchange in the isolated p300 TAZ2 domain, are located adjacent to this patch and it seems likely that some or all of these will form part of the B-Myb-binding surface.

**Figure 7:**
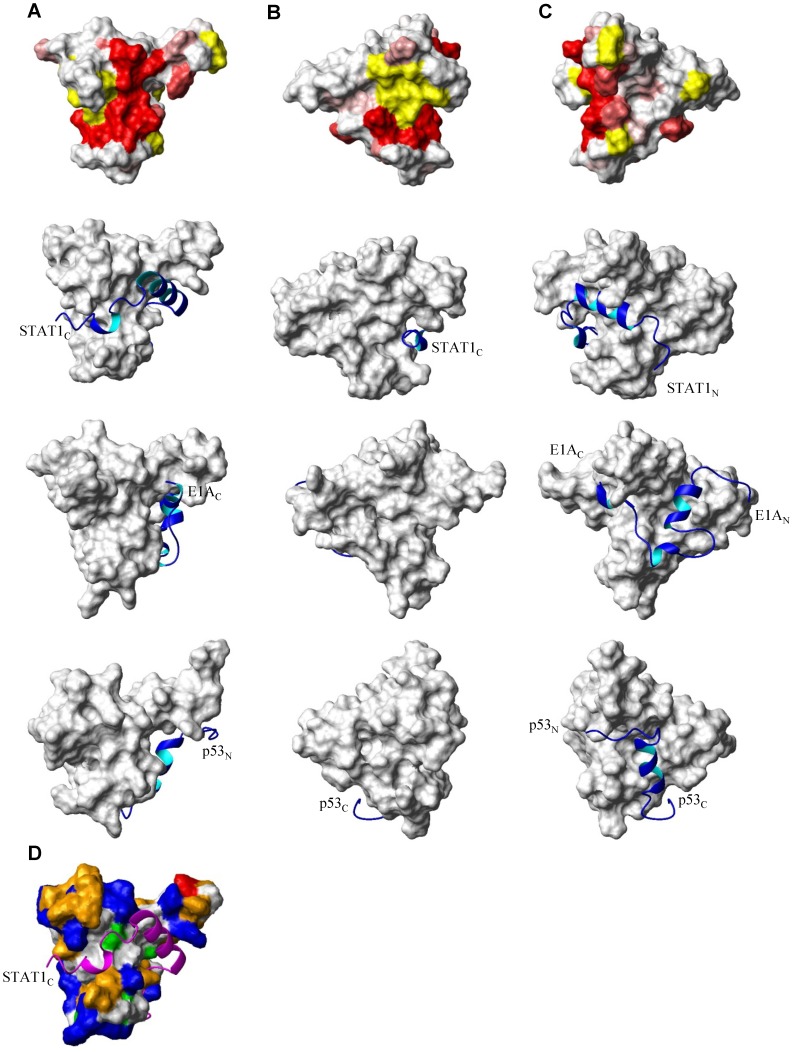
Comparison of the B-Myb, STAT1, E1A and p53 transactivation domain binding sites on p300/CBP TAZ2. Panel A shows a contact surface view of CBP TAZ2 (top) with the location of the B-Myb TAD binding site on p300 TAZ2 highlighted as described in figure 5. For comparison, the structures of STAT1 TAD-CBP TAZ2 (row 2; PDB code 2KA6), E1A CR1-CBP TAZ2 (row 3; PDB code 2KJE) and p53 TAD1-p300 TAZ2 (row 4 PDB code 2K8F) are shown in the same orientation [56], [61], [64], with the TAZ2 domain shown as a contact surface and the STAT1 TAD, E1A CR1 and p53 TAD1 as a ribbon representation of their backbone conformation. Only the well defined residues of STAT1 (721–750), E1A (53–83) and p53 (9–31) that contact TAZ2 are shown in the figure. The views in panels B and C are rotated about the y axis by 90° and −90° compared to panel A. Panel D shows the structure of STAT1 TAD-CBP TAZ2, in the same orientation shown in panel A, with the TAZ2 domain shown as a contact surface and STAT1 TAD as a ribbon representation of the domain. TAZ2 residues are coloured on the basis of residue type, with basic amino acids in blue (Arg, Lys and His), acidic in red (Asp and Glu), polar in orange (Ser, Thr, Asn and Gln), cysteine in green and hydrophobic in white (Trp, Phe, Tyr, Ala, Val, Ile, Leu, Met, Pro and Gly).

**Figure 8:**
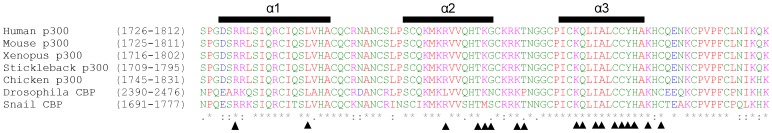
Multiple sequence alignment of the highly homologous TAZ2 domains of p300 and CBP. The multiple sequence alignment of the TAZ2 domain of human, mouse, western clawed frog (Xenopus tropicalis), stickleback and chicken p300, and drosophila and pond snail CBP, illustrates the high degree of sequence homology between the TAZ2 domains of a diverse range of species. Residues are coloured according to the residue type, with small and hydrophobic residues in red (AVFPMILW), acidic residues in blue (DE), basic residues in magenta (RK) and residues containing a hydroxyl, sulfhydryl or sidechain amide group in green (STYHCNQ). Glycine was also coloured in green. Consensus symbols are shown below the sequence. Residues marked with an ‘*’ were fully conserved between sequences. The symbol ‘:’ indicates conservation between groups with strongly similar properties and ‘.’ indicates conservation between groups of weakly similar properties. TAZ2 residues that were significantly shifted upon binding to B-Myb are indicated by triangles shown below the consensus. The positions of the helices in p300 TAZ2, which were identified by analysis of the backbone resonance assignments using the chemical shift index method are indicated above the sequence. The alignment was prepared using ClustalW.

The transactivation domain (TAD) of the transcription factor STAT1 (Signal transducer and activator of transcription-1, residues 710–750) has been shown to interact with essentially the same surface of CBP TAZ2 as reported here for B-Myb TAD (figure 7) [56]. Interestingly, the core of the overlapping B-Myb TAD and STAT1 TAD binding surface on TAZ2 is absolutely conserved over a diverse range of species as is clearly evident in the sequence alignment shown in figure 8. The STAT1 TAD undergoes coupled folding and binding to form one well defined helix in the complex, which, together with extended regions located on either side of the helix, fits into a hydrophobic groove on the surface of TAZ2. This groove is surrounded by a high proportion of positively charged and polar residues, which favours the binding of amphipathic helices and extended regions, such as those seen in the STAT1 TAD (figure 7D). As previously mentioned, the B-Myb TAD has the potential to fold upon binding to form two short amphipathic helices (α1 and α2). The second of these helices (α2) would contain a hydrophobic and an acidic face (figure 6), which would allow it to make complementary interactions with the interaction surface on TAZ2. It seems likely that this predicted helical region (α2), together with either the preceding predicted helix (α1), or the highly conserved acidic/hydrophobic rich region located on the C-terminal side of B-Myb TAD α2 would bind TAZ2 in a similar manner to that seen by the STAT1 TAD (figure S1 and figure 7). This type of interaction would account for the observed shift in B-Myb TAD tryptophan fluorescence. Such an interaction would allow the non-polar residues of B-Myb TAD to make favourable van der Waals contacts with part of the hydrophobic groove in the interaction surface on TAZ2 (figure 7D), whilst the acidic B-Myb TAD side chains could make favourable hydrogen bond and ionic interactions with the basic side-chains of surrounding p300 TAZ2 residues, resulting in the formation of a relatively stable complex.

The structures of TAZ2 in complex with the E1A conserved region 1 (CR1, residues 53–91) and the p53 TAD1 (residues 1–39) have also been solved and are shown in figure 7 [61], [64]. E1A CR1 also fits into a hydrophobic groove on the surface of TAZ2 forming a number of hydrophobic interactions, which are stabilised by complementary ionic interactions between acidic residues of E1A CR1 and surrounding basic residues of TAZ2. The C-terminal half of E1A CR1, binds to the same region of TAZ2 as the amphipathic helix of STAT1, and therefore partially overlaps with the B-Myb TAD interaction surface (figure 7). The p53 TAD1 binding site shows some overlap with the B-Myb TAD interaction surface (figure 7), but this is to a much lesser extent than seen for the STAT1 TAD and E1A CR1, with the main B-Myb TAD and p53 TAD1 interaction surfaces being located on opposite sides of TAZ2. The E1A CR1 has been shown to compete with the p53 TAD1 for binding to TAZ2 [61]. It seems very likely that the B-Myb TAD will also compete with the STAT1 TAD, E1A CR1 and possibly the p53 TAD1 for binding to TAZ2. Given the fact that p300 and CBP are present in limited amounts in cells competition between these transcriptional regulators may play an important role in transcriptional regulation.

During the preparation of this manuscript the ternary structure of p300 TAZ2 in complex with DNA-bound MADS-box/MEF2 domain of MEF2 (residues 1–95) was reported [68]. The structure shows that three MEF2 dimers can simultaneously bind to distinct interaction surfaces on TAZ2, as shown in figure S3. In contrast to the previously discussed TAZ2 binding partners, which are composed of short helical and extended regions that fit into hydrophobic grooves on the surface of TAZ2, each MEF2 dimer interacts with TAZ2 via two parallel helices, which bind discrete surfaces of TAZ2. In addition, no significant structural changes are observed in the MEF2 dimers upon binding to TAZ2. One of the MEF2 dimers (shown in blue in figure S3) binds to the same surface of TAZ2 as both the STAT1 and B-Myb TADs, and would almost certainly compete with these two TADs for binding to TAZ2. A second MEF2 dimer (shown in green) sits adjacent to the STAT1 and B-Myb TAD binding site, whilst the third dimer binds to a distinct surface of TAZ2. The presence of these additional interaction sites would probably allow TAZ2 to simultaneously interact with both MEF2 and B-Myb TAD.

The work reported here provides compelling evidence that B-Myb TAD binds to a specific region on the surface of the TAZ2 domain of p300, which strongly supports the assignment of p300 as a key functional partner of B-Myb *in vivo*. The two domains bind with moderate affinity, which probably reflects the coupled binding and folding of the B-Myb TAD, but clearly favours the formation of a dynamic complex, well suited to producing a transient activation of gene expression.

## Supporting Information

Figure S1 **Multiple sequence alignment of the highly homologous TADs of mouse (mB-Myb), human (hB-Myb), chicken (cB-Myb) and zebrafish B-Myb (zB-Myb).** Residues with absolutely conserved sequence identity are highlighted in red, whilst those with conserved sequence similarity in three or more species are highlighted in yellow. The positions of the two potential helices are indicated above the sequence. The consensus sequence is shown below. Amino acids with absolutely conserved sequence identity are shown in uppercase; those with sequence similarity in over 75% of the sequences are shown in lowercase. Similar residues were grouped as follows: AVILM, FYW, KRH, DE, STNQ, PG and C. The symbol ‘!’ is used to denote either I or V, ‘$’ denotes L or M, ‘%’ denotes F or Y, and ‘#’ denotes any of NDQE. The alignment was prepared using ClustalW and ESPript.cgi (http://npsa-pbil.ibcp.fr/cgi-bin/align_clustalw.pl).
(TIFF)

Figure S2 **Location of the B-Myb TAD binding site on p300 TAZ2.** Panel A shows a ribbon representation of the TAZ2 domain of CBP [30], while panel B shows a contact surface view in the same orientation. In panel C the surface view of CBP TAZ2 has been rotated by 180° about the y axis to reveal the opposite face of the domain. The contact surfaces have been coloured according to the magnitude of the minimal shifts induced in backbone amide resonances of equivalent residues in p300 TAZ2 by binding of the B-Myb TAD. Residues that showed a minimal shift change of less than 0.075 ppm are shown in white, over 0.15 ppm in red, and between 0.075 and 0.15 ppm are coloured according to the level of the shift on a linear gradient between white and red. No chemical shift perturbation data could be obtained for the residues shown in yellow. Panels D-F show the equivalent views of the structure of p300 TAZ2 [67]. The contact surface of p300 TAZ2 is coloured as explained for CBP TAZ2. In addition, the C-terminal 22 residues of the p300 TAZ2 (1813–1834) structure, which are absent from both our p300 TAZ2 construct and the CBP TAZ2 structure (panels A-C) are shown in green.
(TIF)

Figure S3 **Comparison of the B-Myb TAD and the DNA bound MEF2 binding sites on p300 TAZ2.** Panel A shows a contact surface view of CBP TAZ2 (left) with the location of the B-Myb TAD binding site on p300 TAZ2 highlighted as described in figure 5. For comparison, the structure of p300 TAZ2 bound to three MEF2 dimers (right; PDB code; 3P57, residues 1–95 [68]) are shown in the same orientation, with the TAZ2 domain shown as a contact surface and the three MEF2 dimers as ribbon representations of their backbone conformations. For clarity the DNA fragments, which bind to opposite face of the MEF2 dimers have been omitted from the figure. The views in panels B and C are rotated about the y axis by 90° and −90° compared to panel A.
(TIFF)
