# Supplementary figures and images for: Interaction of the Transactivation Domain of B-Myb with the TAZ2 Domain of the Coactivator p300: Molecular Features and Properties of the Complex
Source: PLoS One. 2012 Dec 31;7(12):e52906. doi: 10.1371/journal.pone.0052906 (PMC3534135; doi:10.1371/journal.pone.0052906)

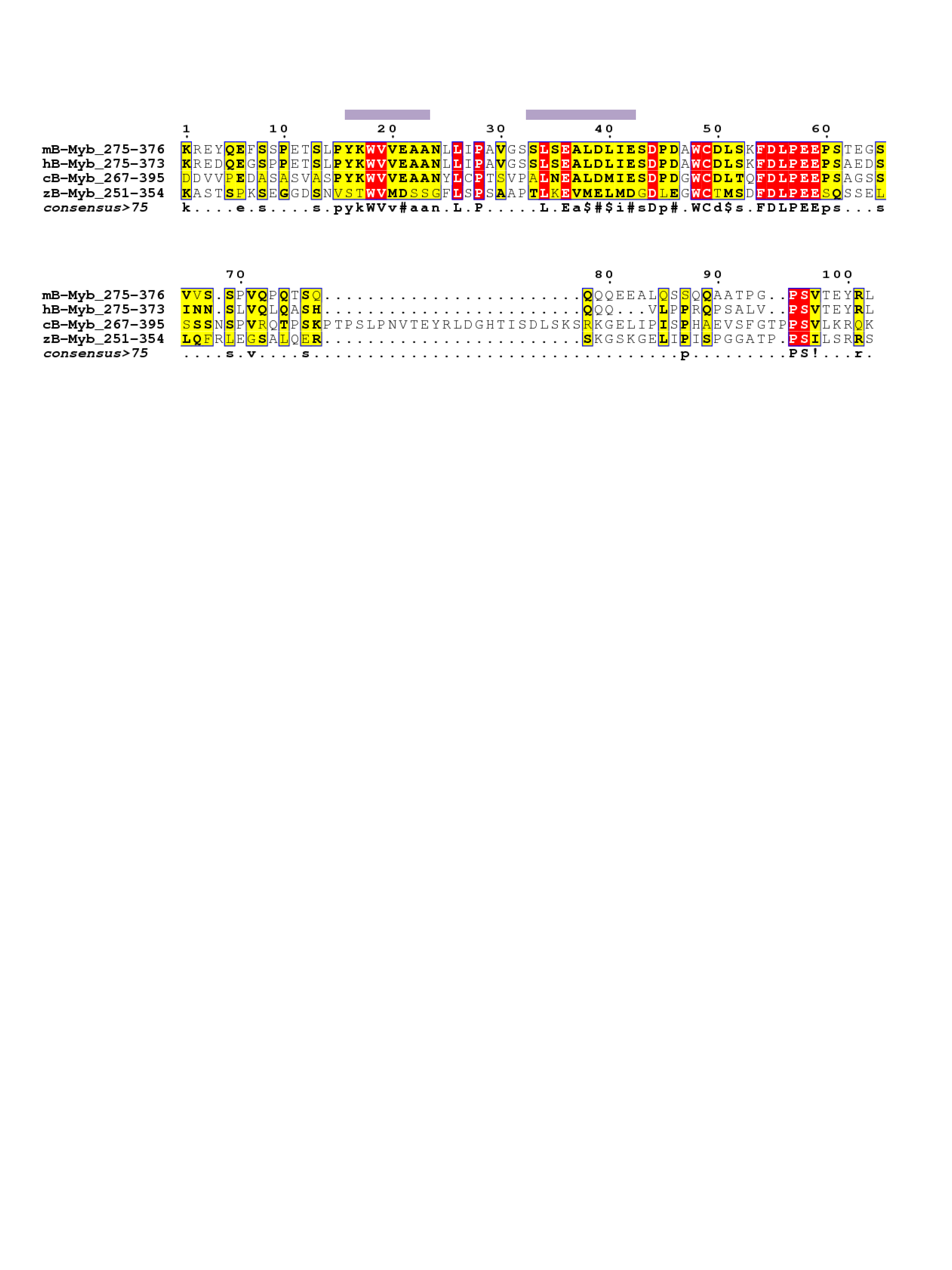

Supplement: Figure S1 — Multiple sequence alignment of the highly homologous TADs of mouse (mB-Myb), human (hB-Myb), chicken (cB-Myb) and zebrafish B-Myb (zB-Myb). Residues with absolutely conserved sequence identity are highlighted in red, whilst those with conserved sequence similarity in three or more species are highlighted in yellow. The positions of the two potential helices are indicated above the sequence. The consensus sequence is shown below. Amino acids with absolutely conserved sequence identity are shown in uppercase; those with sequence similarity in over 75% of the sequences are shown in lowercase. Similar residues were grouped as follows: AVILM, FYW, KRH, DE, STNQ, PG and C. The symbol ‘!’ is used to denote either I or V, ‘$’ denotes L or M, ‘%’ denotes F or Y, and ‘#’ denotes any of NDQE. The alignment was prepared using ClustalW and ESPript.cgi (http://npsa-pbil.ibcp.fr/cgi-bin/align_clustalw.pl). (TIFF) [file pone.0052906.s001.tiff]

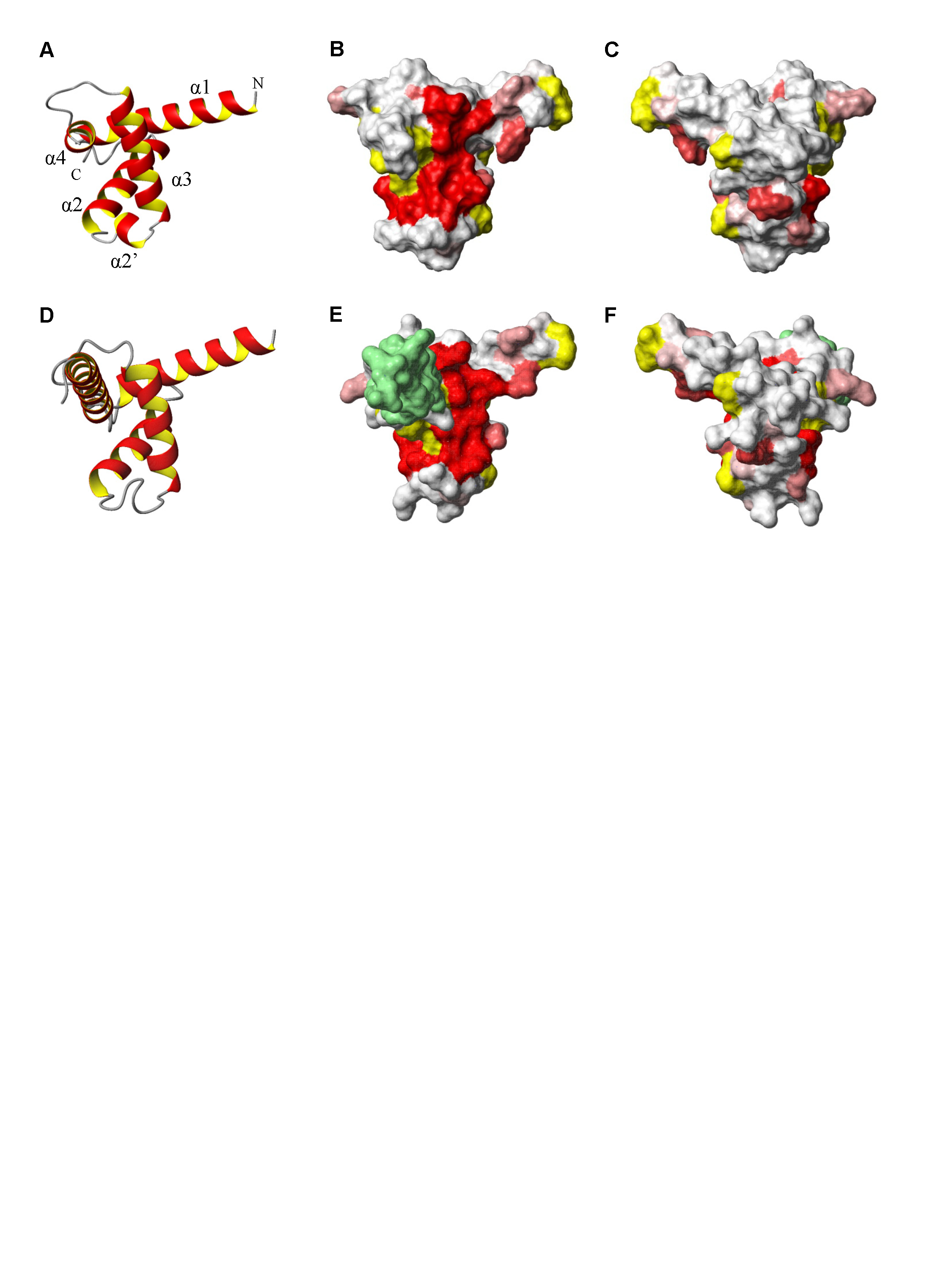

Supplement: Figure S2 — Location of the B-Myb TAD binding site on p300 TAZ2. Panel A shows a ribbon representation of the TAZ2 domain of CBP [30], while panel B shows a contact surface view in the same orientation. In panel C the surface view of CBP TAZ2 has been rotated by 180° about the y axis to reveal the opposite face of the domain. The contact surfaces have been coloured according to the magnitude of the minimal shifts induced in backbone amide resonances of equivalent residues in p300 TAZ2 by binding of the B-Myb TAD. Residues that showed a minimal shift change of less than 0.075 ppm are shown in white, over 0.15 ppm in red, and between 0.075 and 0.15 ppm are coloured according to the level of the shift on a linear gradient between white and red. No chemical shift perturbation data could be obtained for the residues shown in yellow. Panels D-F show the equivalent views of the structure of p300 TAZ2 [67]. The contact surface of p300 TAZ2 is coloured as explained for CBP TAZ2. In addition, the C-terminal 22 residues of the p300 TAZ2 (1813–1834) structure, which are absent from both our p300 TAZ2 construct and the CBP TAZ2 structure (panels A-C) are shown in green. (TIF) [file pone.0052906.s002.tif]

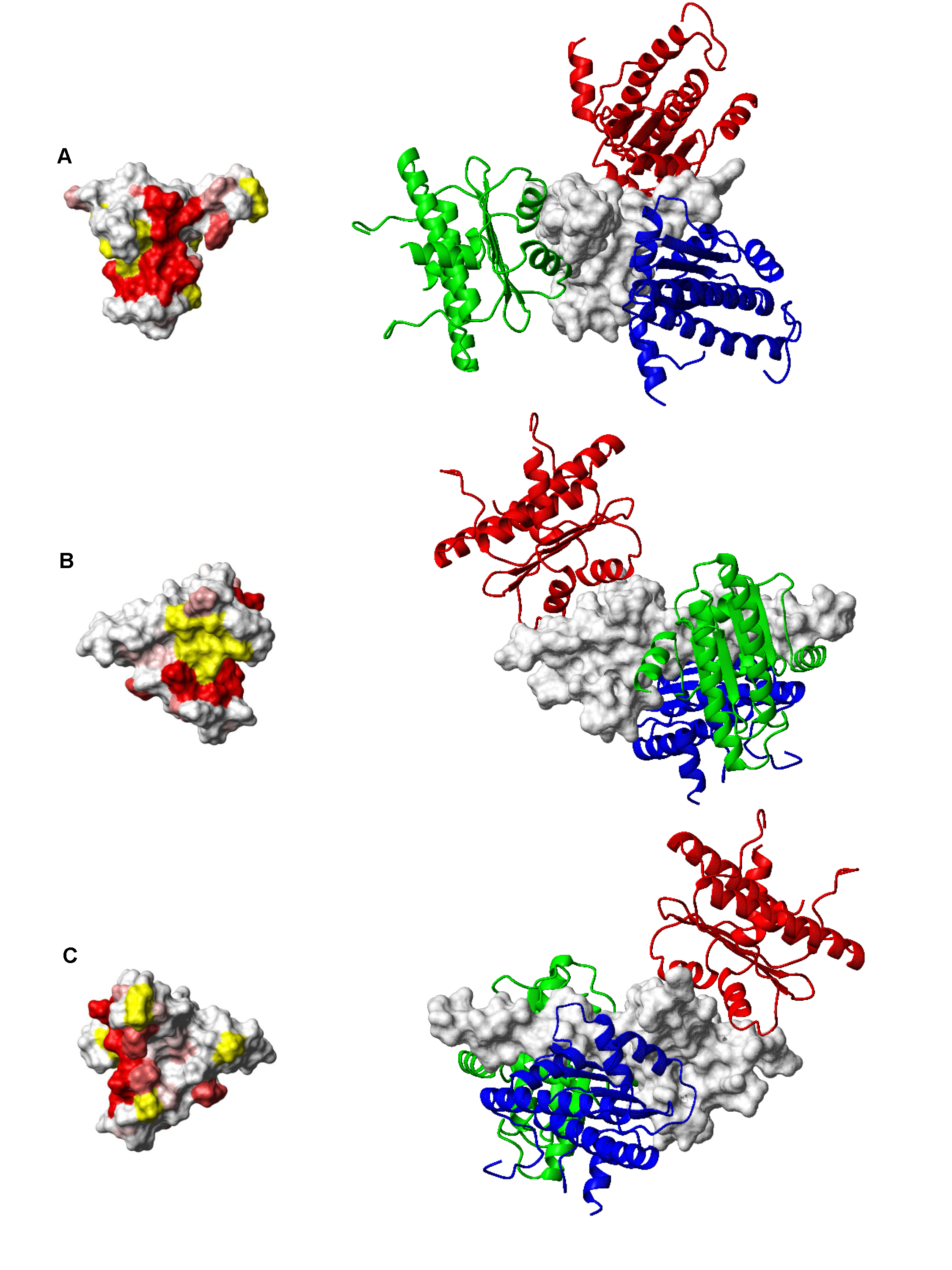

Supplement: Figure S3 — Comparison of the B-Myb TAD and the DNA bound MEF2 binding sites on p300 TAZ2. Panel A shows a contact surface view of CBP TAZ2 (left) with the location of the B-Myb TAD binding site on p300 TAZ2 highlighted as described in figure 5. For comparison, the structure of p300 TAZ2 bound to three MEF2 dimers (right; PDB code; 3P57, residues 1–95 [68]) are shown in the same orientation, with the TAZ2 domain shown as a contact surface and the three MEF2 dimers as ribbon representations of their backbone conformations. For clarity the DNA fragments, which bind to opposite face of the MEF2 dimers have been omitted from the figure. The views in panels B and C are rotated about the y axis by 90° and −90° compared to panel A. (TIFF) [file pone.0052906.s003.tiff]
